# A Review of the Clinical Presentation, Causes, and Diagnostic Evaluation of Increased Intracranial Pressure in the Emergency Department

**DOI:** 10.5811/westjem.18500

**Published:** 2024-09-06

**Authors:** Cristiana Olaru, Sam Langberg, Nicole Streiff McCoin

**Affiliations:** Ochsner Health, Department of Emergency Medicine, New Orleans, Louisiana

## Abstract

Increased intracranial pressure (ICP) is encountered in numerous traumatic and non-traumatic medical situations, and it requires immediate recognition and attention. Clinically, ICP typically presents with a headache that is most severe in the morning, aggravated by Valsalva-like maneuvers, and associated with nausea or vomiting. Papilledema is a well-recognized sign of increased ICP; however, emergency physicians often find it difficult to visualize the optic disc using ophthalmoscopy or to accurately interpret digital fundus photographs when using a non-mydriatic retinal camera.

Emergency ultrasound can evaluate the optic nerve sheath diameter (ONSD) and optic disc elevation to determine whether increased ICP is present, however, the studies have been small with different definitions and measurements of the ONSD. The ONSD threshold values for increased ICP have been reported anywhere from 4.8 to 6.3 millimeters.

Neuroimaging is the next step in the evaluation of patients with papilledema or high clinical suspicion of increased ICP, as it can identify most structural causes or typical radiological patterns of increased ICP. Neuroradiographic signs of increased ICP can be helpful in suggesting idiopathic intracranial hypertension (IIH), especially when papilledema is absent.

Patients with papilledema and normal neuroimaging may undergo lumbar puncture as part of their clinical workup. The cerebrospinal fluid (CSF) opening pressure remains one of the most important investigations to establish the diagnosis of IIH. A CSF evaluation is also required to exclude other etiologies of elevated ICP such as infectious, inflammatory, and neoplastic meningitis. Invasive ICP measurement remains the standard to measure and monitor this condition.

## INTRODUCTION

Increased intracranial pressure (ICP), regardless of etiology, is a life-threatening condition that requires prompt diagnosis and treatment. It can lead to decreased cerebral perfusion pressure with subsequent cerebral ischemia and herniation, and thus potential disability and increased mortality.^1^ Recognition of elevated ICP is of utmost importance in the emergency department (ED). Knowledge of the clinical presentation (which can help differentiate not only between multiple causes of headache and altered mental status but also between causes of elevated ICP) and diagnostic options and their accuracy is paramount for correct diagnosis and rapid treatment.

The Monro-Kellie hypothesis states that the sum of the intracranial volume of blood, brain, cerebrospinal fluid (CSF), and other components (eg, tumor, hematoma) is constant. The skull is a rigid container; hence, an increase in one of the intracranial components will cause a decrease in the volume of one or more of the other components. Intracranial blood (especially in the venous compartment) and CSF are the two components whose volume can adapt most easily to accommodate an increase in the volume of intracranial contents. When the compensatory capacity is exhausted, the ICP begins to rise, compromising cerebral perfusion and causing cerebral ischemia or herniation. Normal ICP is 7–15 millimeters of mercury (mmHg) or 10–20 centimeters of water (cmH_2_O) in adults.^1^ ^,^ ^2^

Cerebral perfusion pressure (CPP)=Mean arterial pressure (MAP)−intracranial pressure (ICP).

## CAUSES AND CLINICAL PRESENTATION

Increased ICP is caused by a variety of disease processes such as space-occupying lesions (eg, mass, hemorrhage); obstructive hydrocephalus; communicating hydrocephalus (eg, inadequate reabsorption of CSF such as seen in subarachnoid hemorrhage secondary to hypersecretion of CSF and fibrosis of arachnoid granulations); venous outflow obstruction (eg, cerebral venous sinus thrombosis); diffuse cerebral edema (eg, vasogenic, as seen in tumors; cytotoxic, as seen in traumatic brain injury or stroke; interstitial, as seen in hydrocephalus or meningitis; or osmotic, such as seen in hyponatremia, diabetic ketoacidosis); increased CSF secretion (eg, choroid plexus tumor); and idiopathic causes (eg, idiopathic intracranial hypertension [IIH]).^3^ ^,^ ^4^ ^,^ ^5^

The combination of headache, papilledema, and vomiting is considered indicative of increased ICP, although there is no consistent relation between severity of symptoms and the degree of elevated ICP.^2^ Headache is a common complaint in the ED, representing 2.6% of ED visits, and the sixth most common reason for presentation to the ED.^6^ The headache related to increased ICP is typically a global headache commonly described as throbbing or bursting, is most severe in the morning, often aggravated by maneuvers that increase ICP (eg, Valsalva-like maneuvers, coughing, sneezing, recumbency), and is associated with nausea or vomiting.^3^ Other signs and symptoms of increased ICP include changes in behavior such as irritability and restlessness, visual changes (eg, diplopia, visual field deficits), pupillary changes (dilated unreactive, mid-position fixed, pinpoint pupils), bilateral ptosis, impaired upward gaze, focal neurologic deficits, depressed consciousness, seizures, and the ominous findings of decorticate or decerebrate posturing and Cushing triad (bradycardia, hypertension, and respiratory depression).

**Figure 1.**
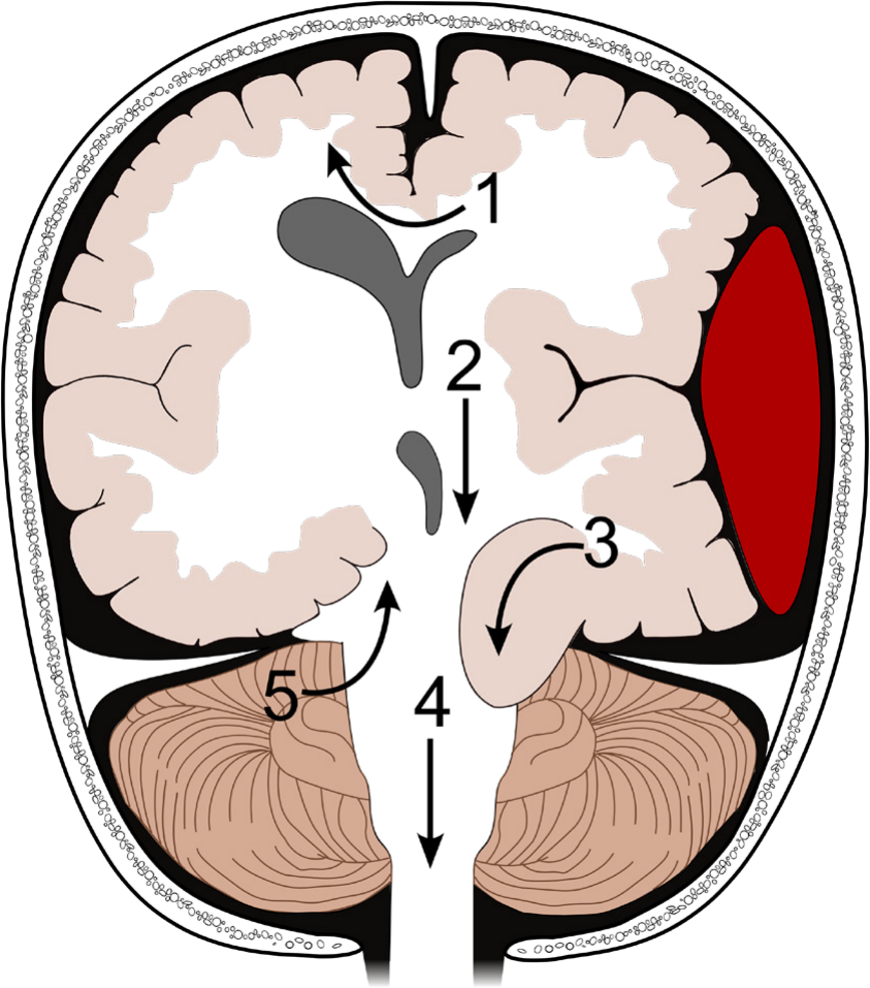
Types of brain herniation^11^: 1) subfalcine herniation; 2) central descending transtentorial herniation; 3) lateral descending transtentorial herniation; 4) tonsillar herniation; and 5) ascending cerebellar transtentorial herniation. Adapted from User: Delldot, CC BY-SA 3.0 <http://creativecommons.org/licenses/by-sa/3.0/>, version 21:08, 5 March 2008 via Wikimedia Commons.

A progressive deterioration in level of consciousness can be seen with worsening increased ICP (except for IIH, which is characterized by normal mental status).^2^ Brain herniation leads to further brain injury and ischemia, compression of vessels and cranial nerves, and obstruction of the normal circulation of CSF producing hydrocephalus. (See Figure 1 for types of brain herniation.) Owing to its location, each type of herniation is associated with specific neurologic findings.^7^

Subfalcine herniation, also known as midline shift or cingulate herniation, is the most common type of herniation. It is usually caused by mass effect that pushes the ipsilateral cingulate gyrus down and under the falx cerebri. The quantification of the midline shift is made by measuring the deviation of the septum pellucidum compared to midline at the level of the foramen of Monro. This measurement is used for prognosis with less than 5 millimeters (mm) of deviation associated with good prognosis and greater than 15 mm associated with poor prognosis. It can present with hypobulia, apathy, and indifference. If the anterior cerebral artery is compressed, it will manifest with contralateral or bilateral leg weakness and acute urinary retention.^7^

Descending transtentorial herniation is the second most common type of cerebral herniation. It occurs when brain tissue is displaced downward through the tentorial notch and may be lateral (anterior and posterior) or central. Lateral hernias involve the medial temporal lobe; anterior hernias involve the uncus (also called uncal herniation); and posterior hernias involve the parahippocampal gyrus. In the central hernia, there is descent of the diencephalon, midbrain, and pons.^7^

Anterior descending transtentorial (uncal) herniation leads to compression of the parasympathetic fibers running with the third cranial nerve, causing an ipsilateral fixed and dilated pupil. Compression of the ipsilateral cerebral peduncle will cause contralateral motor paralysis since the motor tract fibers cross below this level; however, the contralateral cerebral peduncle can be compressed against the edge of the tentorium causing a false localizing sign with ipsilateral hemiparesis.^8^ Posterior descending transtentorial herniation is due to herniation of the parahipoccampal gyrus, presenting with symptoms of Parinaud syndrome (vertical gaze palsy, loss of pupillary reflex to light with preservation of pupillary constriction with convergence, upper eyelid retraction, convergence-retraction nystagmus).^9^ ^,^ ^10^ Central descending transtentorial herniation is due to herniation of the thalamus and midbrain through the tentorial notch and the medulla though the foramen magnum. It causes acute obstructive hydrocephalus and posterior cerebral artery injury, clinically presenting with agitation followed by obtundation, bilaterally poorly reactive or potentially midpoint fixed pupils, and then decorticate followed by decerebrate posturing, Cushing triad, coma, and death.^9^

Ascending cerebellar transtentorial herniation is due to cerebellar herniation superiorly through the tentorial notch. It presents with symptoms of pontomedullary compression: obtundation; cardiorespiratory instability; severe bradycardia; arrhythmia; abd pinpoint pupils.^9^ Tonsillar herniation involves herniation of the tonsils of the cerebellum through the foramen magnum into the upper spinal canal, compressing the medulla. This may result in cardiorespiratory impairment, hypertension, high pulse pressure, and Cheyne-Stoke respiration. The combination of bradycardia, hypertension, and irregular respirations is known as Cushing’s reflex and occurs in approximately one third of cases of tonsillar herniation.^2^ It may also cause pinpoint pupils, flaccid paralysis, and sudden death.

Idiopathic intracranial hypertension, formally known as pseudotumor cerebri or benign intracranial hypertension, deserves special attention as it has a unique presentation. It is a syndrome of increased ICP of unclear etiology that occurs most often in obese women of childbearing age (average age 28 years). It occurs less often in men (approximately 9%) who are usually obese and average 37 years of age at diagnosis.^12^ It is usually a diagnosis of exclusion that is characterized by signs and symptoms of increased ICP, normal mental status, and absence of focal neurologic signs (although it can be associated with sixth and seventh nerve palsies). Neuroimaging might show signs of elevated CSF pressure but without obstruction or deformity of the ventricles and without identifiable cause of the increased ICP. The CSF evaluation will have opening pressure greater than 25 cmH2O but with normal CSF composition.^3^

Common symptoms of IIH are headache, visual disturbances, and pulsatile tinnitus. The most common presenting symptom of IIH is headache, which is found in 84% of the patients. The headache is constant, non-pulsating, exacerbated by coughing or Valsalva maneuver, and it has a progressive course. The second most frequent symptom of IIH is visual disturbance such as variable, visual field defects that commonly go unnoticed by the patient until severe; transient visual obscurations (transient unilateral or bilateral visual loss lasting less than one minute and often precipitated by postural changes, with full rapid visual recovery to baseline); enlarging blind spots; diminished visual acuity in patients with advanced disease; diplopia, especially in the horizontal plane generally due to sixth cranial nerve palsy; or blurry vision due to shortening of the globe secondary to increased ICP. Tinnitus, another common symptom in IIH, is more often bilateral, pulsatile, synchronous with heart rate, and can occur with variable frequency from daily to monthly.^3^ ^,^ ^12^ Idiopathic intracranial hypertension is characterized by normal mental status; however, it can cause disabling headaches and blindness.

## DIAGNOSTIC EVALUATION

### Papilledema

The presence of optic nerve head edema (ONHE) in patients with headache signifies a secondary cause for the headache and the need for further urgent evaluation.^13^ This condition is commonly encountered in papilledema (optic disc swelling due to increased ICP); optic neuropathy (optic neuritis, ischemic optic neuropathy); and pseudopapilledema (disc elevation without nerve fiber layer edema). The distinction between the three major causes of disc swelling is based on history, eye examination including fundoscopy, and ancillary testing. Other causes of optic disc swelling are central retinal vein occlusion, diabetic papillopathy, uveitis, optic disc tumors, malignant hypertension, and optic nerve infiltration (such as seen in sarcoidosis, lymphoma, and leukemia).^14^ Optic neuropathies lead to a more severe visual loss and are usually sudden, unilateral, and associated with afferent pupillary defect and impaired color vision.^14^

Pseudopapilledema is associated with optic nerve variants that mimic papilledema ophthalmoscopically, such as congenital abnormalities; crowded hyperopic disc; optic disc hamartomas; or optic nerve head drusen. Visual loss may occur, but it is more indolent, painless, and frequently unnoticed by the patient. Pseudopapilledema is stable over time compared to untreated papilledema, which will change and progress in time. There are also ophthalmoscopic findings that will help differentiate papilledema from pseudopapilledema.^14^ Optic disc drusen are acellular deposits located in the optic nerve head. In children, the optic disc drusen are not calcified; they resemble papilledema with optic nerve head swelling and can be difficult to diagnose on opthalmoscopy.^15^ With age, the optic disc drusen become calcified and easier to diagnose on ophthalmoscopy and ultrasound, as optic disc drusen are hyperechoic with posterior acoustic shadowing.^15^

The Frisén classification is the most frequently used papilledema grading system and describes stages of optic disc swelling (grades 0–5); however, it has poor inter-rater reliability. Therefore, more descriptive terminology is often used to describe papilledema (eg, mild vs high grade).^14^ ^,^ ^16^ ^,^ ^17^ Papilledema is usually bilateral and symmetrical; however, it can be asymmetrical and, rarely, it can be unilateral or even absent.^12^ ^,^ ^18^ Papilledema is thought to be secondary to either axoplasmic stasis that causes axonal swelling or due to enlargement of the subarachnoid space. Usually, the development of papilledema requires at least 1–5 days of persistently elevated ICP; however, it has also been found to develop rapidly, in hours, in subarachnoid and intraparenchymal hemorrhages. If the elevated ICP is treated, papilledema usually resolves over weeks to months.^14^

Non-expert clinicians often find it difficult to properly view the optic disc using ophthalmoscopy.^19^ Phase I of the FOTO-ED study found that direct ophthalmoscopy was rarely and inadequately performed by emergency physicians (EP) in a large academic medical center where EPs failed to diagnose any cases of optic-nerve head edema using direct ophthalmoscopy.^20^ Phase II of the FOTO-ED study found that non-mydriatic retinal photography in the ED was superior to direct ophthalmoscopy performed by EPs; however, EPs do not commonly perform this and identified only 16 of 35 relevant findings (sensitivity 46%).^21^ Sachdeva et al^13^ performed a cross-sectional analysis of patients with ONHE in the prospective FOTO-ED study and found that 2.6% of patients presenting to the ED with a chief complaint of headache, acute vision loss, focal neurologic deficit, or a diastolic blood pressure ≥120 mmHg had ONHE. The most common final diagnoses were IIH (19/37), CSF shunt malfunction/infection (3/37), and optic neuritis (3/37), thus reiterating the importance of ocular fundus examination in these patients.^13^

### Emergency Ultrasound Evaluation for Increased Intracranial Pressure

**Figure 2.**
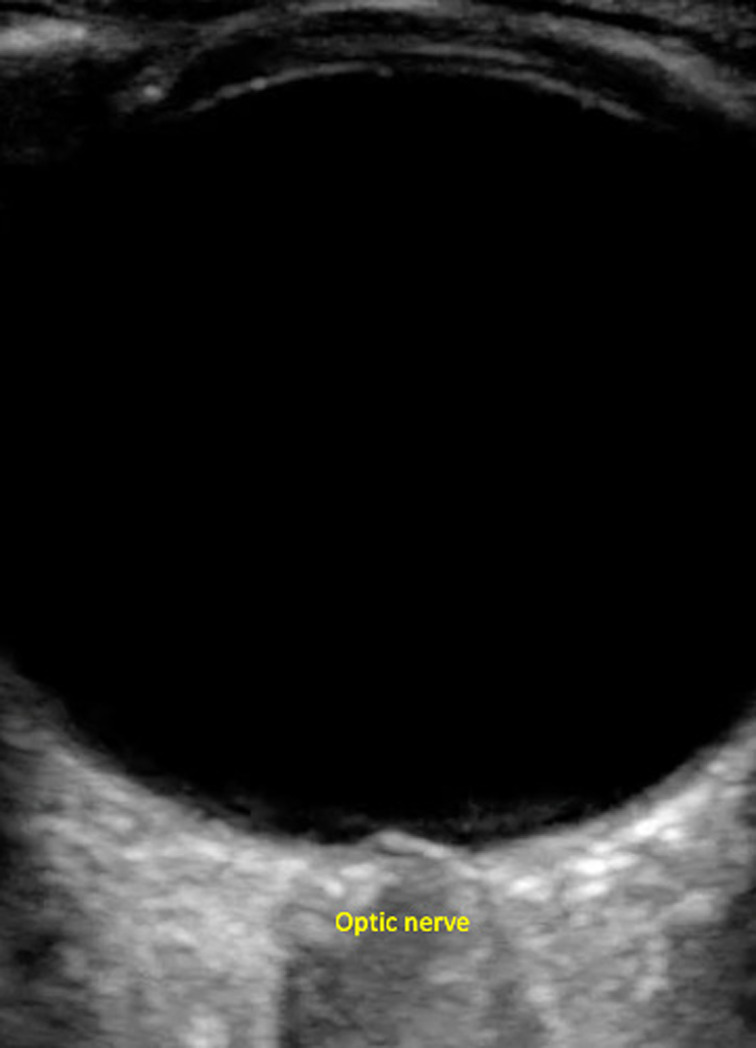
Optic nerve appearance on ultrasound.

Emergency ultrasound is an easy-to-use, noninvasive method of increased ICP assessment by evaluating the optic nerve sheath diameter (ONSD) and the optic disc elevation (ODE). The optic nerve can be thought of as an outpouching of intact brain tissue with the intraorbital component CSF and fluctuates in size based on changes in ICP. Increased ICP causes enlargement of the subarachnoid space and increase of the ONSD. The bulbous portion of the optic nerve, approximately 3 mm posterior to the globe, appears to be the most distensible and sensitive to changes in ICP.^22^ On ultrasound, the globe appears as a round, anechoic structure. The optic nerve presents as a hypoechoic structure posterior to the globe (see Figure 2).

#### Optic disc elevation

**Figure 3.**
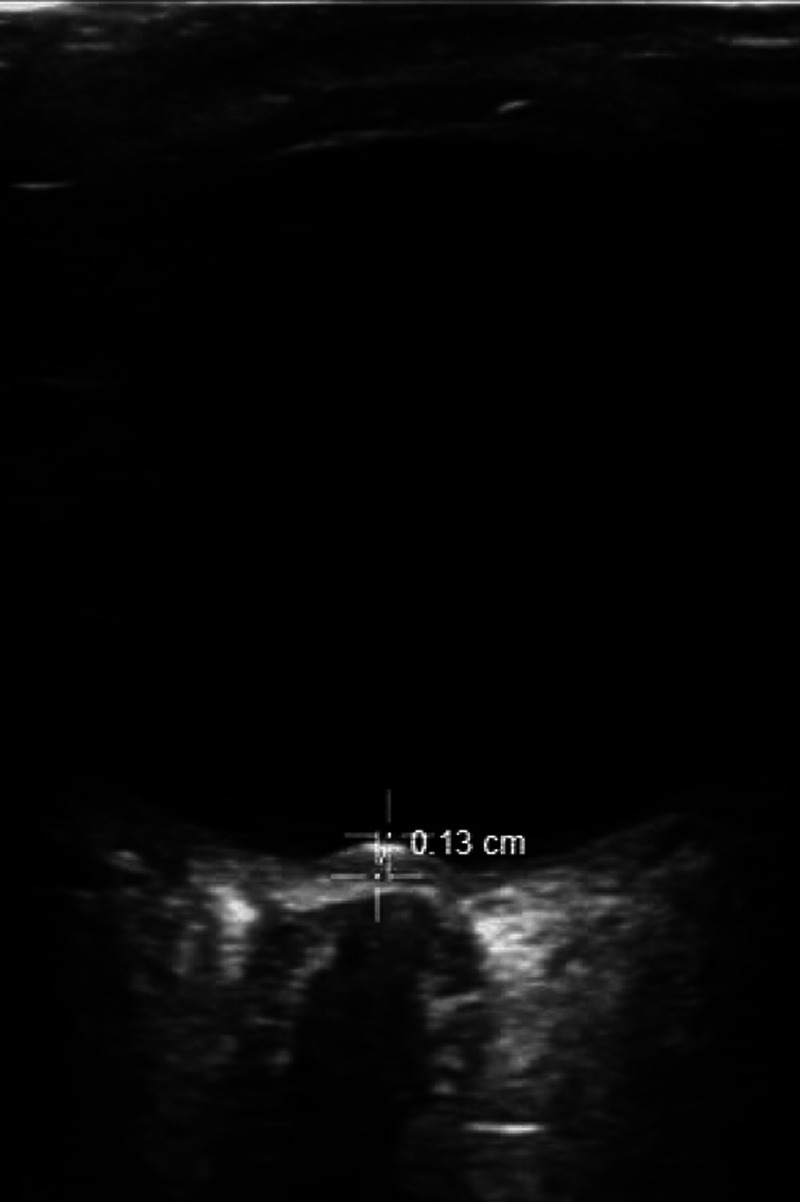
Optic disc elevation (ODE) measurement between the top of the swollen optic disc and the lamina cribrosa. In this figure, ODE of 0.13 cm suggests optic disc edema.

Optic disc elevation refers to the height of elevation of the optic disc from the lamina cribrosa (the area through the sclera where the optic nerve axons pass).^23^ The measurement is performed with the optic nerve in the horizontal plane, and the view with the maximum disc elevation is selected. Disc elevation is measured from the uppermost part of the swollen disc to the strongly reflecting line representing the lamina cribrosa (see Figure 3).^24^ Teismann et al^25^ determined that a cutoff value of 0.6 mm for optic disc elevation, as measured by ultrasound, predicted the presence of optic disc edema noted on fundoscopic exam with a sensitivity of 82% (95% confidence interval [CI] 48–98%) and a specificity of 76% (95% CI 50–93%). A cutoff value of 1.0 mm yielded a sensitivity of 73% (95% CI 39–94%) and specificity of 100% (95% CI 81–100%). In this study, most patients had IIH causing disc swelling due to elevated ICP; however, disc swelling can also be found in patients with multiple sclerosis, infiltrative processes such as sarcoidosis or lymphoma, infections directly affecting the optic nerve, and microvascular infarction caused by malignant systemic hypertension.

#### Optic nerve sheath diameter

**Figure 4.**
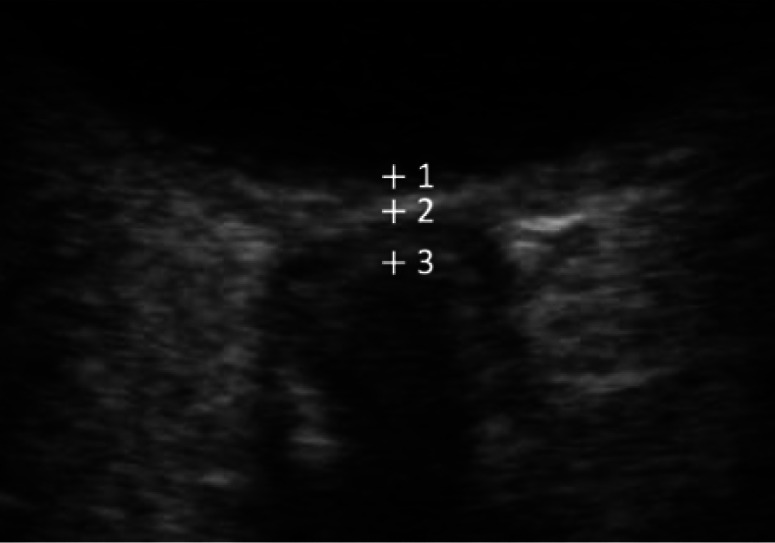
Optic nerve sheath diameter superficial caliper placement: 1) retina level; 2) lamina cribrosa level; and 3) top of the hypoechoic optic nerve level.

The ONSD is measured 3 mm deep to the globe where it appears to be the most distensible and sensitive to changes in ICP.^22^ Three possible positions for depth markers have been described in studies: 1) location where imaginary nerve midline intersects the contour of the retina; 2) hyperechoic reflection corresponding to the lamina cribrosa; and 3) top of the hypoechoic structure corresponding to the optic nerve (see Figure 4). However, these discrepancies did not affect ONSD values, most likely because the distance between the different anatomical landmarks used is less than 1 mm resulting in comparable ONSD values. Stevens et al^26^ recommends using the papilla as reference for the 3-mm depth assessment.

**Figure 5.**
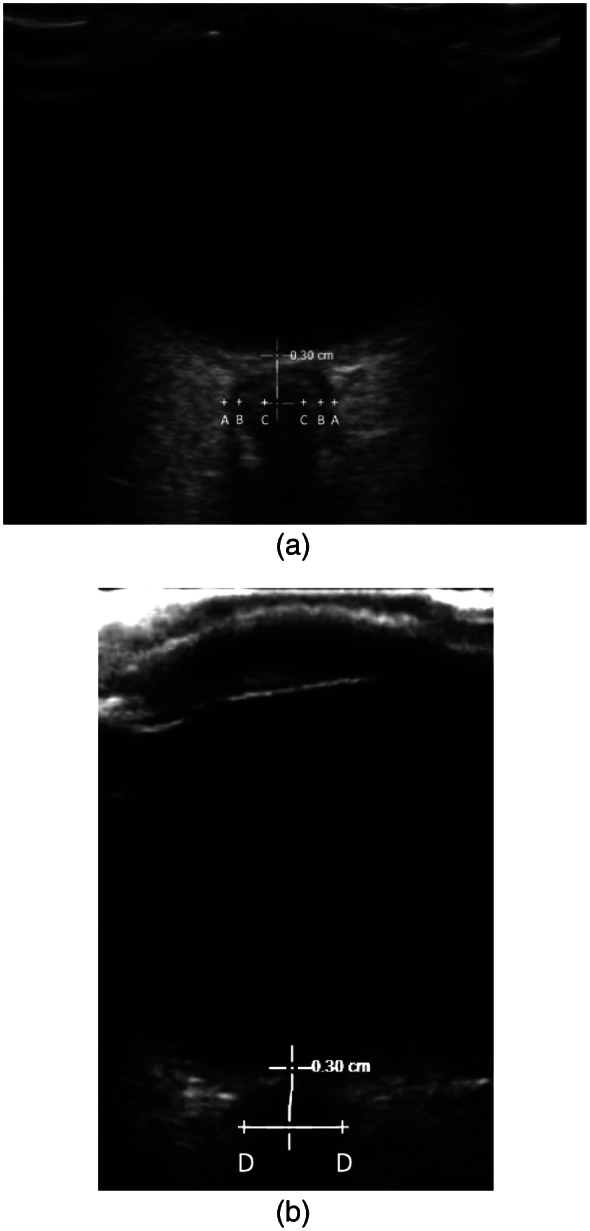
(a) Optic nerve (between C calipers) as one anechoic band surrounded by two hyperechoic striped bands (between C and B calipers on each side of the optic nerve) between the hyperechoic retrobulbar fat (A calipers). (b) Optic nerve with one single anechoic band representing the optic nerve and its sheath, D calipers at the border between the optic nerve sheath and the retrobulbar fat.

The optic nerve itself has a diameter of 3 mm, and the optic nerve sheath has a thickness of approximately 1 mm. From the inside out, the sheath consists of pia mater, the subarachnoid space, the arachnoid mater, and the dura mater. When studies were reviewed, two types of images with different echoic characteristics of the optic nerve sheath were described. One group of images showed two hyperechoic, striped bands between the hyperechoic retrobulbar fat (see Figure 5a), while the other group showed a single dark linear structure surrounded by hyperechoic retrobulbar fat (see Figure 5b).^26^

The striped bands have been interpreted to represent either the pia, both pia and dura mater, or the subarachnoid space. Stevens et al^26^ found that C caliper position was the least sensitive to changes in the ICP and corresponds to the outline of the optic nerve itself. B caliper measurement increases in patients with elevated ICP; hence, it incorporates the arachnoid space, and it likely corresponds to the outer edge of the subarachnoid space. B caliper position and calipers measuring a single dark linear structure (D calipers) were found to be equally sensitive to changes in ICP and were recommended as reliable ONSD measurements.^26^

The ONSD threshold values that optimized sensitivity and specificity for elevated ICP varied in studies from 4.8 mm (Rajajee et al^27^) to 6.3 mm (del Saz-Saucedo et al^28^). The studies on ONSD have been small and heterogeneous, and these studies have done the following:

• used different definitions and measurements of ONSD^26^
• used different planes to measure ONSD: only transverse planes^27^ ^–^ ^32^; transverse and sagittal planes^33^ ^–^ ^35^; transverse and coronal^36^
• predominantly used mean values of the ONSD measurements obtained from both eyes, with Agrawal et al^36^ identifying the highest transverse measurement and Rajajee et al^27^ studying each individual transverse measurement
• enrolled different patient populations, with multiple studies enrolling neurocritical care patients with traumatic and non-traumatic brain injury, and some studies enrolling clinic patients with IIH^28^ ^,^ ^32^
• used different confirmations of elevated ICP including computed tomography (CT),^37^ ^,^ ^38^ lumbar puncture (LP),^28^ ^,^ ^32^ or external ventricular drain (EVD) and intraparenchymal catheter^33^ ^,^ ^36^
• defined elevated ICP as either 20 mmHg or 25 cmH_2_O (20 mmHg = 27 cmH_2_O^39^).

In a meta-analysis including 18 prospective studies that had ICP measured by EVD or intraparenchymal catheter (LP measurements excluded), Aletreby et al^40^ demonstrated that ONSD showed reasonable accuracy in diagnosing raised ICP. The highest sensitivity was achieved using an ONSD cutoff of more than 6 mm.^40^ While ONSD of <5 mm correlates with normal ICP, the cutoff ONSD for elevated ICP varies from study to study with >6 mm demonstrating a high sensitivity for elevated ICP. An ODE >0.6 mm can also be helpful in determining papilledema; however, ODE can be seen in multiple other medical conditions. While ONSD and ODE can be helpful in screening for papilledema, larger studies with standardized ONSD measurements and a consistent ICP cutoff are still needed. It is also known that papilledema can be asymmetrical; hence, each eye ONSD measurements should be studied independently.

### Neuroimaging

In the evaluation of a patient with increased ICP, neuroimaging has two main purposes: to exclude structural causes; and to identify typical radiographic patterns of elevated ICP. Non-enhanced CT is commonly used as the first test for evaluation of secondary headaches and altered mental status in the emergency setting, and it is the standard imaging modality in acute head trauma. Computed tomography is widely available, has shorter acquisition time, and is less expensive than brain magnetic resonance imaging (MRI). In the emergency setting, CT is regularly performed to readily identify conditions that require surgical intervention.^7^ A CT can identify brain edema, tumors, hydrocephalus, intracranial hemorrhage, and signs of cerebral herniation. A CT may also detect signs of increased ICP, such as ventricular or sulcal effacement, compression of the basal CSF cisterns, herniation, or midline shift. However, ICP can be elevated even in the setting of a normal CT.

Compared to CT, MRI provides better tissue characterization, especially for posterior fossa disease, and is required for evaluation of underlying brain lesions. Brain MRI with contrast can identify most structural causes of increased ICP, and magnetic resonance venography (MRV) with contrast will exclude cerebral venous sinus thrombosis.^3^ In IIH, brain MRI and MRV are important to exclude secondary causes of elevated ICP. Additionally, empty sella turcica, posterior flattening of the globe, enlargement of the perioptic subarachnoid space, optic nerve midportion tortuosity, hyperintensity of the optic nerve and optic disc, and bilateral transverse sinus stenosis have been found to be associated with IIH; and the sensitivity and specificity improve with a combination of these neuroimaging signs.^12^ ^,^ ^41^ Slit-like ventricles, tight subarachnoid space, and inferior position of the cerebellar tonsils shows low sensitivity but good specificity of 90–97% for IIH.^12^

### Intracranial Pressure Measurement

Patients with papilledema and normal neuroimaging could undergo LP as part of their clinical workup to measure the ICP in search for etiologies such as IIH. The CSF opening pressure remains one of the most important findings to establish a diagnosis of IIH. The CSF pressure may vary considerably with time; thus, the possibility to repeat LP or perform a more invasive ICP monitoring should be considered if clinical suspicion is high.^12^ A CSF evaluation is also required to exclude other etiologies of elevated ICP such as infectious, inflammatory, or neoplastic meningitis.^3^ The LP is obtained in lateral recumbent position, while the CSF opening pressure should be measured with the legs extended, head in neutral position, and the patient breathing normally. The normal CSF opening pressure in adults is 10–20 cmH_2_O and is considered high if greater than 25 cmH_2_O. A CSF opening pressure of 20–25 cmH_2_O is considered borderline, and it is interpreted in the clinical context.^3^

Idiopathic intracranial hemorrhage without papilledema is well recognized. These patients tend to have lower opening pressure levels than those who present with papilledema. In the absence of papilledema, the other criteria should be met, with the additional symptom of unilateral or bilateral abducens nerve palsies. If there is no abducens nerve palsy, three of the following neuroimaging criteria should be met: 1) empty sella; 2) flattening of the posterior aspect of the globe; 3) distention of the subarachnoid space with or without a tortuous optic nerve; and 4) transverse sinus stenosis. A normal opening pressure level does not exclude the diagnosis of IIH when the patient has other typical symptoms. Conversely, an increased opening pressure level without appropriate symptoms should not be interpreted as IIH.^42^

A LP carries no risk of herniation if there is no brain shift, whether CSF pressure is raised or not and whether papilledema is present or not. A CT is used to diagnose brain shift seen in space-occupying lesions and diffuse brain swelling. Findings of brain shift on CT may demonstrate the following: displacement of brain structures; loss of differentiation between gray and white matter; flattened gyri; effacement of CSF spaces (such as sulci, Sylvian fissures, ventricles and basal cisterns); dilated ventricles (in obstructive pathology); and in advanced stages herniation (displacement of brain from one intracranial compartment to another).^43^

An invasive ICP measurement device such as external ventricular drain or intraparenchymal device remains the standard to determine the pressure in the cranial vault. The necessity for ICP measurement is either deduced by a pathological CT or a consciousness impairment score of ≤8 on the Glasgow Coma Scale.^44^

## CONCLUSION

Increased intracranial pressure represents a life-threatening diagnosis. Idiopathic intracranial hemorrhage, while not life-threatening, can cause irreversible visual loss and disabling headaches. Clinical presentation requires immediate recognition and investigation. While papilledema is important to diagnose in the ED, emergency physicians have found it difficult to diagnose by direct ophthalmoscopy.^20^ Ocular ultrasound (optic nerve sheath diameter and optic disc elevation) is commonly used to screen for increased ICP; however, there is need for further research using standardized ONSD measurements. Neuroimaging remains the first step in investigating elevated ICP as it excludes structural causes and identifies typical radiological patterns of elevated ICP. Invasive ICP measurement remains the standard to measure and monitor ICP.
